# Plasmacytoid DCs From Patients With Sjögren's Syndrome Are Transcriptionally Primed for Enhanced Pro-inflammatory Cytokine Production

**DOI:** 10.3389/fimmu.2019.02096

**Published:** 2019-09-04

**Authors:** Maarten R. Hillen, Aridaman Pandit, Sofie L. M. Blokland, Sarita A. Y. Hartgring, Cornelis P. J. Bekker, Eefje H. M. van der Heijden, Nila H. Servaas, Marzia Rossato, Aike A. Kruize, Joel A. G. van Roon, Timothy R. D. J. Radstake

**Affiliations:** ^1^Laboratory of Translational Immunology, University Medical Center Utrecht, Utrecht University, Utrecht, Netherlands; ^2^Department of Rheumatology and Clinical Immunology, University Medical Center Utrecht, Utrecht University, Utrecht, Netherlands; ^3^Department of Biotechnology, University of Verona, Verona, Italy

**Keywords:** Sjögren's syndrome, plasmacytoid dendritic cells, type-I interferon, transcriptomics, gene network analysis

## Abstract

Primary Sjögren's syndrome (pSS) is a systemic auto-immune disease typified by dryness of the mouth and eyes. A majority of patients with pSS have a type-I interferon (IFN)-signature, which is defined as the increased expression of IFN-induced genes in circulating immune cells and is associated with increased disease activity. As plasmacytoid dendritic cells (pDC) are the premier type-I IFN-producing cells and are present at the site of inflammation, they are thought to play a significant role in pSS pathogenesis. Considering the lack of data on pDC regulation and function in pSS patients, we here provided the first in-depth molecular characterization of pSS pDCs. In addition, a group of patients with non-Sjögren's sicca (nSS) was included; these poorly studied patients suffer from complaints similar to pSS patients, but are not diagnosed with Sjögren's syndrome. We isolated circulating pDCs from two independent cohorts of patients and controls (each *n* = 31) and performed RNA-sequencing, after which data-driven networks and modular analysis were used to identify robustly reproducible transcriptional “signatures” of differential and co-expressed genes. Four signatures were identified, including an IFN-induced gene signature and a ribosomal protein gene-signature, that indicated pDC activation. Comparison with a dataset of *in vitro* activated pDCs showed that pSS pDCs have higher expression of many genes also upregulated upon pDC activation. Corroborating this transcriptional profile, pSS pDCs produced higher levels of pro-inflammatory cytokines, including type-I IFN, upon *in vitro* stimulation with endosomal Toll-like receptor ligands. In this setting, cytokine production was associated with expression of hub-genes from the IFN-induced and ribosomal protein gene-signatures, indicating that the transcriptional profile of pSS pDCs underlies their enhanced cytokine production. In all transcriptional analyses, nSS patients formed an intermediate group in which some patients were molecularly similar to pSS patients. Furthermore, we used the identified transcriptional signatures to develop a discriminative classifier for molecular stratification of patients with sicca. Altogether, our data provide in-depth characterization of the aberrant regulation of pDCs from patients with nSS and pSS and substantiate their perceived role in the immunopathology of pSS, supporting studies that target pDCs, type-I IFNs, or IFN-signaling in pSS.

## Introduction

Primary Sjögren's syndrome (pSS) is a systemic rheumatic autoimmune disease characterized by mononuclear infiltration of the exocrine glands, associated with dysfunction and destruction of the glands and dryness of primarily mouth and eyes (1). Patients experience debilitating fatigue, can suffer from systemic manifestations that can involve a wide range of organs, and have an increased chance of lymphoma (2). At this time, no effective treatment is available and a better understanding of immunopathology is needed to provide novel therapeutic targets.

An interferon (IFN)-signature, defined as the increased expression of a panel of type-I IFN induced genes (IFIG; e.g., *MX1, IFIT3, IFI44*), is present in the majority of pSS patients and is associated with increased systemic disease activity (3, 4). Type-I IFNs stimulate monocytes to produce increased levels of B cell activating factor (BAFF), an important mediator in pSS that drives B cell activation and auto-antibody production (4). In addition, deficiency for the type-I IFN receptor in mice prevents experiment Sjögren's syndrome (5). As such, type-I IFNs are considered to be crucial mediators in pSS pathogenesis (6, 7).

Plasmacytoid dendritic cells (pDCs) are uniquely capable of rapidly producing high levels of type-I IFN upon activation. pDCs constitutively express endosomal Toll-like receptors (TLR) 7 and 9, through which they recognize viral RNA and DNA but can also bind endogenous nucleic acids. Triggering of these TLRs results in production of type-I IFNs or other pro-inflammatory cytokines, depending on the cellular compartment in which the receptors encounter their ligand (8). Importantly, type-I IFNs also act on pDCs themselves, and autocrine or paracrine IFN primes them for enhanced IFN-production and potently amplifies their responses (9–11). As self-nucleic acids, in the form of autoantibody-complexes and apoptotic cell material, are present in patients with pSS and can strongly activate pDCs via TLR7 and TLR9, these cells are thought to be a major source of type-I IFN in pSS (12–14).

In patients with pSS, numbers of pDCs in peripheral blood are decreased and associated with increased salivary gland inflammation (15, 16). Furthermore, pDCs infiltrate the pSS salivary glands and local pDC numbers correlate with the number of IFN-α producing cells (15, 17, 18). Moreover, pDCs from IFN signature-positive pSS patients have increased expression of TLR7 and RNA-sensing receptors, identifying pathways via which these cells can be activated to produce enhanced type-I IFNs (19). Thus, a considerable body of evidence supports an important role for pDCs in pSS pathogenesis. Despite this, there is a lack of data on purified pDCs from pSS patients, at least in part due to their scarcity (<0.5% of leukocytes). In addition, pDCs could possibly play a role in (a subset of) patients with non-Sjögren's sicca (nSS): this poorly studied group suffers from unexplained dryness similar to patients with pSS, but is not diagnosed with Sjögren's syndrome by the clinician and does not meet the classification-criteria. Comparing the transcriptional profile of pSS and nSS pDCs with those from healthy donors can provide a better understanding of their role in pathogenesis and aid in molecular patient stratification (20).

## Materials and Methods

### Patients and Controls

All pSS patients met the 2002 AECG classification-criteria (21). nSS patients presented with dryness-complaints without a known cause, did not have any generalized autoimmune disease including pSS as evaluated by an experienced rheumatologist, and did not fulfill the classification-criteria for pSS. Fourteen nSS patients were clinically re-evaluated several years after initial sample collection; labial biopsy, assessment of laboratory parameters, and physical examination were all performed at follow-up and the 2016 ACR-EULAR criteria (22) were used for classification. For *in vitro* experiments, independent donors were included (Table 1). The medical ethics committee of the UMC Utrecht approved the study (METC nr 13-697), all patients gave their written informed consent.

**Table 1:** Donor characteristics.

	**RNA sequencing**						***In vitro*** **experiments**	
	**Discovery cohort (*****n*** **=** **31)**			**Replication cohort (*****n*** **=** **31)**				
	**HC**	**nSS**	**pSS**	**HC**	**nSS**	**pSS**	**HC**	**pSS**
N [M/F]	8 [0/8]	9 [0/8]	14 [3/11]	9 [0/9]	11 [0/11]	11 [0/11]	17 [0/17]	22 [2/20]
Age (year)	58 [54–59]	43 [34–61]	54 [44–61]	51 [48–55]	45 [37–60]	55 [40–63]	54 [47–57]	54 [47–68]
LFS (foci/4 mm^2^)	–	0.0 [0.0–0.0]	1.9 [1.1–3.0]	–	0.1 [0.0–0.5]	2.2 [1.5–3.0]	–	2.0 [1.3–3.0]
ESSDAI	–	–	2 [1–6]	–	–	5 [3–11]	–	5 [2–10]
ESSPRI	–	–	4 [2–7]	–	–	5 [3–7]	–	6 [4–7]
Schirmer (mm/5 min)	–	3 [1–14]	5 [2–15]	–	8 [3–18]	12 [6–23]	–	3 [0–8]
ANA (no. positive [%])	–	1 [11%]	10 [71%]	–	5 [45%]	9 [82%]	–	18 [82%]
SSA (no. positive [%])	–	2 [22%]	8 [57%]	–	3 [27%]	7 [64%]	–	17 [77%]
SSB (no. positive[%])	–	0 [%]	4 [29%]	–	0 [0%]	3 [27%]	–	12 [55%]
Serum IgG (g/L)	–	12 [8–14]	14 [10–18]	–	13 [10–14]	18 [10–33]	–	14 [8–17]
ESR (mm/hour)	–	11 [7–15]	11 [6–25]	–	8 [6–15]	14 [8–34]	–	17 [7–34]
CRP (mg/L)	–	1 [0–4]	1 [1–7]	–	1 [0–2]	2 [1–3]	–	2 [1–6]
Treatment (no. positive [%])	–	1 [11%]	3 [21%]	–	1 [10%]	4 [36%]	-	5 [23%]

*There was no overlap in donors between any of the cohorts investigated. Values are Median [IQR] unless stated otherwise. LFS, Lymphocyte focus score; ESSDAI, EULAR Sjögren's syndrome disease activity index; ESSPRI, EULAR Sjögren's syndrome patient reported index; ANA, Anti-nuclear antibodies; SSA, Anti-SSA/Ro; SSB, Anti-SSB/La; ESR, Erythrocyte sedimentation rate; CRP, C-reactive protein. Immunosuppressive treatment included (combinations of) hydroxychloroquine (n = 10), prednisone (n = 4), azathioprine (n = 5)*.

### Cell Isolation and RNA Isolation

Peripheral blood mononuclear-cells were isolated from heparinized peripheral blood by density centrifugation using Ficoll-Paque Plus (GE Healthcare). pDCs were freshly isolated by MACS using BDCA-4+ isolation kit (Miltenyi Biotec). In addition, monocytes were freshly isolated by MACS from a subset of 43 donors (14 HC, 10 nSS, 19 pSS; randomly selected from either cohort) using the CD14+ isolation kit (Miltenyi) after isolation of pDCs to quantify the IFN-score. Cells were lysed in RLTplus buffer (Qiagen Allprep Universal kit) supplemented with beta-mercaptoethanol (final concentration 1%) for transcriptional analyses. Total RNA was purified using AllPrep Universal Kit (Qiagen), according to the manufacturer's instructions. RNA concentration was assessed with Qubit RNA Kit (Thermo Fisher Scientific) and RNA integrity was measured by capillary electrophoresis using a Bioanalyzer (Agilent) and the RNA 6000 Nano Kit (Agilent Technologies); all samples had RIN-score >7.0.

### RNA Sequencing and Analysis

For the discovery cohort samples, RNA sequencing was performed at the Beijing genomics institute on a NextSeq 500 sequencer (Illumina) by applying standard manufacturer's protocols. About 20 million paired-end (91 bp) reads were generated for each sample. For the replication cohort, RNA sequencing was performed at the Beijing genomics institute using an Illumina HiSeq 4000 sequencer (Illumina), by applying standard manufacturer's protocols. About 20 million paired-end (100 bp) reads were generated for each sample. Raw reads obtained from RNAseq were quality checked using the FastQC tool (https://www.bioinformatics.babraham.ac.uk/projects/fastqc/). All samples passed the quality check. For each sample, reads were then aligned to the human genome assembly (GRCh38 build 79) (23) using STAR aligner (24). The aligned reads (mapping quality > 30) were used to calculate the read counts using Python package HTSeq (25) for each annotated gene. Since samples were collected, isolated and stored at different timepoints and by different individuals, this can create batch effects in the RNASeq data. We used RUVSeq (26) to remove unwanted variance (k = 1 parameter) for both cohorts and used RUVSeq-corrected read counts for further analysis. To approximate the biological variability and overdispersion found in the RNASeq data, the batch-corrected read counts were modeled as a negative binomial distribution to identify differentially expressed genes (DEGs) using Bioconductor/R package DESeq2 (27). We used Wald's test to identify DEGs in each pair-wise comparison performed between the three groups (HC, nSS, and pSS) and used likelihood ratio test (LRT) to identify DEGs considering multiple groups. Differences in gene expression with a nominal *p*-value of < 0.05 were considered differentially expressed. Variance stabilizing transformation was applied to the raw read count data to obtain normalized gene counts (variance stabilized data or VSD), which were used for subsequent analyses.

To investigate if the pSS pDCs were similar to TLR-activated cells, we compared their gene expression profiles with the expression signature of *in vitro* stimulated pDCs using gene set enrichment analysis (GSEA) (28). For this, we used a publically available RNA-sequencing dataset of TLR-ligand stimulated isolated pDCs (29). Bioconductor/R package FGSEA was used to perform the GSEA analysis and compare the genes differentially-expressed in the pSS pDCs to those differential upon stimulation with pRNA or CPG-P, which was done separately for the up and down-regulated genes (30). Ten thousand permutations were performed to calculate the significance of enrichment scores and FDR-corrected *p*-values.

### Weighted Gene Co-expression Network Analysis (WGCNA)

To study the inter-dependence between genes and their interactions, we constructed two gene co-expression networks (one for the discovery cohort and another for the replication cohort) using WGCNA Bioconductor/R package (31). The gene co-expression networks were constructed using all the genes that were differentially expressed (p <0.05) either in the pairwise analysis (Wald's test) or in the multi-group comparison (LRT). The modules were defined by first performing an unsigned pair-wise spearman's correlation between genes and subsequently transforming the co-expression similarities into an adjacency matrix (that provides the relative connection strengths between genes) and scaling the adjacency matrix to achieve a scale-free topology (scaling power = 6, selected to have a network that fits the scale-free topology criterion).

For each module the gene or the first principal component was calculated and plotted using ggplot2 and ggsci libraries in R. For each gene, the connectivity represents the sum of connection strengths (or the weighted correlations) of each node with its neighbors in a given module. We normalized the connectivities for each module in the range [0, 1], where 1 represents the maximum and 0 represents the minimum connectivity in the module. We calculated the overlap between the modules constructed from the two independent cohorts and used Fisher's exact test to identify modules with significant overlaps (32). We performed pathway enrichment analysis on replicated DEGs found in modules exhibiting significant overlap in the two cohorts using R/Bioconductor package ReactomePA (33). The networks were plotted using Cytoscape (version 3.6.1) (34).

### pDC Culture and Cytokine Analysis

Isolated pDCs were cultured in RPMI Glutamax (Gibco) supplemented with penicillin, streptomycin (both Gibco), and 10% FBS (Sigma). Cells were cultured at 6^*^10^5^ cells/mL in 96 wells round-bottomed plates (Thermo Fisher Scientific) in the presence of 1 mM of loxoribine (TLR7L; InvivoGen) or 1 μM of CPG-C (TLR9L; InvivoGen). After 3 h, supernatants were harvested and cells lysed, samples were stored at −80°C. Cytokines were measured in the supernatants using multiplex immunoassay.

### Quantitative PCR

Quantitative-PCRs were performed in duplicate per sample using SYBR Select Master Mix (Applied Biosystems) and the Quantstudio system (Thermo Fisher Scientific). In the cultured pDCs, mRNA expression was measured and normalized to the mean expression of two housekeeping genes: *GAPDH* and *GUSB* (Supplementary Table 1). The relative fold change (FC) of each sample was calculated in relation to the ΔCt of a random unstimulated sample in the HC group (reference) according to the formula FC = 2^−ΔΔCt^, where ΔΔCt = ΔCt sample—ΔCt reference.

To determine the IFN-score, the relative expression of five IFN-induced genes (*IFI44L, IFI44, IFIT3, LY6E*, and *MX1*) was assessed using qPCR. Expression of each sample was normalized to the mean expression of two housekeeping genes: *GAPDH* and *GUSB* (Supplementary Table 1). The IFN-score was calculated as previously described (4) and divided by the number of genes measured (five).

### Flow Cytometry

To confirm consistent purity of isolated cells, the isolated cell fraction was stained and analyzed using a FACS Canto II or LSRFortessa flow cytometer (both BD). The purity of pDCs was (median [IQR]) 92% [87–95%], monocyte purity was 97% [95–98%]; there were no differences in purity between any of the groups. For assessment of CCR5-expression on pDCs, 1 million PBMCs were stained for 30 min at 4°C and the expression of CCR5 on lineage-negative (CD3-CD14-CD16-CD19-CD56-) and HLA-DR+CD123+BDCA-2+ pDCs was determined (Supplementary Table 2).

### Prediction Model

We used the RUVSeq Bioconductor/R package (26) to consider deviance in the residuals (*k* = 1) from a GLM regression of the counts to batch-corrected read counts from discovery and replication cohorts. We used the caret Bioconductor/R package to perform backward feature selection and 10-fold cross validation to get high prediction accuracy. This allowed us to select 467 genes. We further used the SVM algorithm (svmRadial method from caret R package) to develop a classifier using the 467 selected genes to discriminate pSS patients from HC donors.

### Statistical Analysis

For RNA-sequencing analysis, we used Wald's test to identify DEGs in each pair-wise comparison performed between the three groups (HC, nSS, and pSS) and used LRT to identify DEGs considering multiple groups. For the *in vitro* experiments, differences between patients and controls were assessed using Mann-Whitney *U*-test. Changes in expression of ribosomal proteins after stimulation were analyzed using Wilcoxon matched-pairs signed rank test. Correlations were assessed using Spearman's rank correlation coefficient. Two-sided testing was used for all analyses. Differences and correlations were considered to be significant at *p* < 0.05. As the gene signatures identified were replicated in an independent cohort, no FDR-correction was used for the RNA-sequencing analysis.

## Results

### pSS and nSS pDCs Are Transcriptionally Distinct From HC pDCs and Analogous to TLR-Stimulated Cells

**Figure 1:**
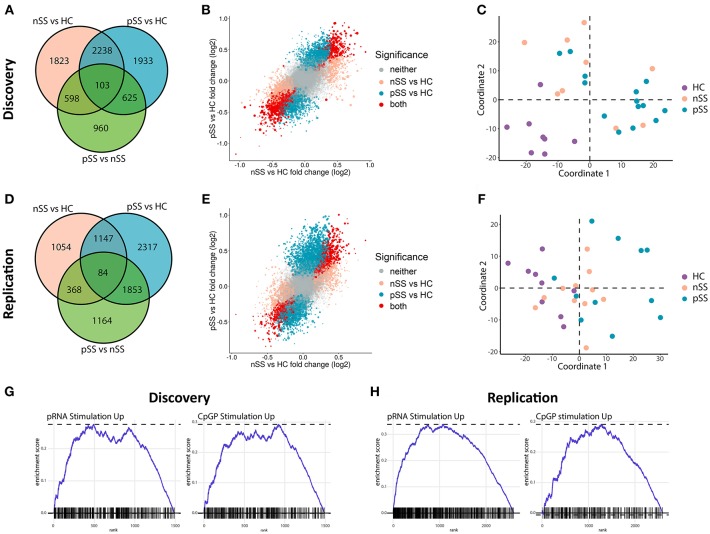
Circulating pDCs from pSS and nSS patients are transcriptionally altered and display gene-expression patterns similar to TLR-stimulated pDCs. pDCs were isolated from peripheral blood of two independent cohorts; discovery and replication (each *n* = 31). RNA sequencing was performed independently for both cohorts. Genes differentially expressed (nominal *p*-value *p* < 0.05) between any of the three groups from the discovery cohort were identified **(A)**. The association between gene expression in pSS vs. HC and nSS vs. HC was plotted; each gene is represented as a dot, with the color reflecting the comparison in which it is significantly differentially expressed and the size reflecting the expression level (larger dots depict genes with higher expression) **(B)**. Multi-dimensional scaling was used to plot the dissimilarities between the donors from the discovery cohort based on the expression of all differentially expressed genes, taking into account all comparisons **(C)**. The same analyses were performed on RNA sequencing data from the replication cohort **(D–F)**. Using gene-set enrichment analysis, the differentially-expressed upregulated genes in pSS pDCs as compared to HC were compared to those of *in vitro* stimulated pDCs from a published study (29). There was a significant overlap in upregulated genes between pDCs stimulated with either pRNA (TLR7 ligand) and CPG-P (TLR9 ligand) and pSS pDCs (all FDR-corrected *p* < 0.05; **G,H**).

We recruited two independent cohorts of patients and healthy controls (discovery and replication, each *n* = 31; Table 1) and compared the transcriptome of their circulating pDCs. The discovery cohort revealed a large number of differentially expressed genes (DEGs) between pSS pDCs and HC pDCs (Figure 1A). Similarly, in the replication cohort we observed a large number of DEGs between pSS and HC (Figure 1D). nSS pDCs exhibited alterations concordant with pSS pDCs in both cohorts, and a substantial fraction of DEGs in pSS pDCs were also differentially expressed in nSS pDCs (47% and 23% in discovery and replication cohort, respectively) (Figures 1A,D). In addition, genes differentially expressed in both pSS and nSS pDCs mostly exhibited the same directionality (>99% of DEG in discovery and replication). However, the magnitude of differences in expression was generally larger in pSS pDCs compared to nSS pDCs (Figures 1B,E). As such, we also observed a substantial number of DEGs between pSS and nSS patients (Figures 1A,D). Multivariate analysis using DEGs from either cohort corroborated this observation; the transcriptional profiles of pDCs from pSS and nSS patients were distinct from HCs, but not necessarily from each other (Figures 1C,F).

Given the observed large transcriptional changes and the fact that pSS is a systemic disease, we hypothesized that the transcriptome of pSS pDCs indicated systemic activation. To test this, we compared the alterations identified in pSS pDCs with publically available transcriptional data from pDCs activated with ligands for TLR7 and TLR9 (29). Gene set enrichment analysis confirmed that a relatively large number of DEGs found in pSS pDCs was also differentially expressed in the stimulated pDCs (Figures 1G,H, Supplementary Figure 1). Thus, the alterations in circulating pDCs from pSS patients are consistent with those found in activated cells.

**Figure 2:**
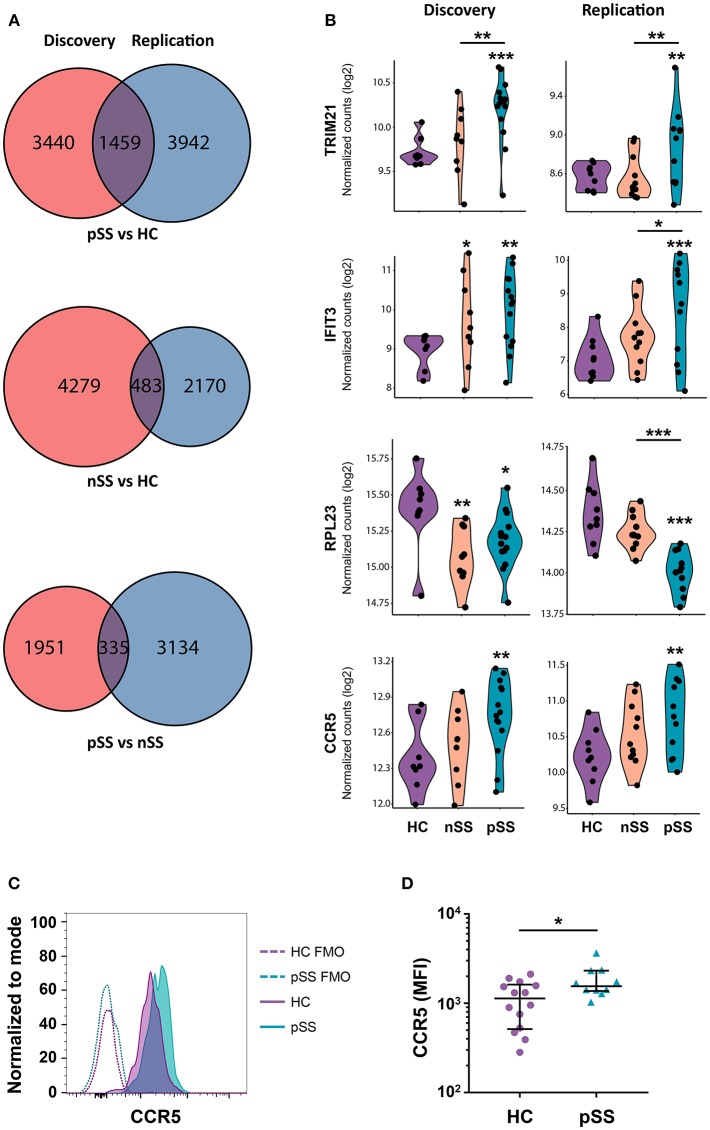
A large set of genes is consistently differentially expressed in patients and CCR5 is increased on the surface of pSS pDCs. Venn diagrams showing the genes differentially expressed (nominal *p*-value < 0.05) in the discovery and replication cohort for the three group comparisons **(A)**. Violin plots of a selection of differentially expressed genes are depicted; expression is shown in normalized read counts, which is plotted on a 2log axis. **(B)**. Mononuclear cells were isolated from peripheral blood of HC and pSS donors and the expression of CCR5 on the surface of lineage-negative and HLA-DR^+^CD123^+^BDCA-2^+^ pDCs from pSS patients (*n* = 10) and HC (*n* = 14) was assessed using flow-cytometry. A representative histogram from one HC and pSS donor is depicted in **(C)**, fluorescence minus one (FMO) controls are indicated with a dotted line. Median fluorescence intensity (MFI) is plotted **(D)**. ^*^, ^**^, and ^***^ represent *p* < 0.05, *p* < 0.01, and *p* < 0.001, respectively.

To identify the genes most robustly and consistently altered, those differentially expressed in both cohorts were selected (Figure 2A). As material from the two cohorts was independently collected, sequenced, and analyzed, we considered these genes to be replicated. Examples of replicated genes are depicted in Figure 2B. These include upregulation of *TRIM21/Ro52*, which is the target of Sjögren's-specific autoantibodies; the decreased expression of a large set of ribosomal proteins (RPs) including *RPL23*; and the increased expression of C-C chemokine receptor 5 (*CCR5*), which is an important driver of pDC migration under inflammatory conditions (35). Validation of this latter observation by assessment of the surface-expression of CCR5 using flow-cytometry confirmed the increased expression of CCR5 on pSS pDCs compared to HCs (Figures 2C,D). In addition, a large number of IFN-induced genes including *IFIT3* was upregulated in pSS and, to a lesser extent, nSS patients, including most genes previously reported to be differentially expressed in pDCs from IFN-signature positive pSS patients (19) (Figure 2B, Supplementary Figure 2). Furthermore, we observed consistent upregulation of *TLR2, TLR4*, and *TLR8* in pSS (Supplementary Figure 2). In general, changes in single genes were most clear in pSS while the nSS patients displayed an intermediate phenotype.

### Network Analysis Identifies Functional Signatures of Differentially Expressed Genes

**Figure 3:**
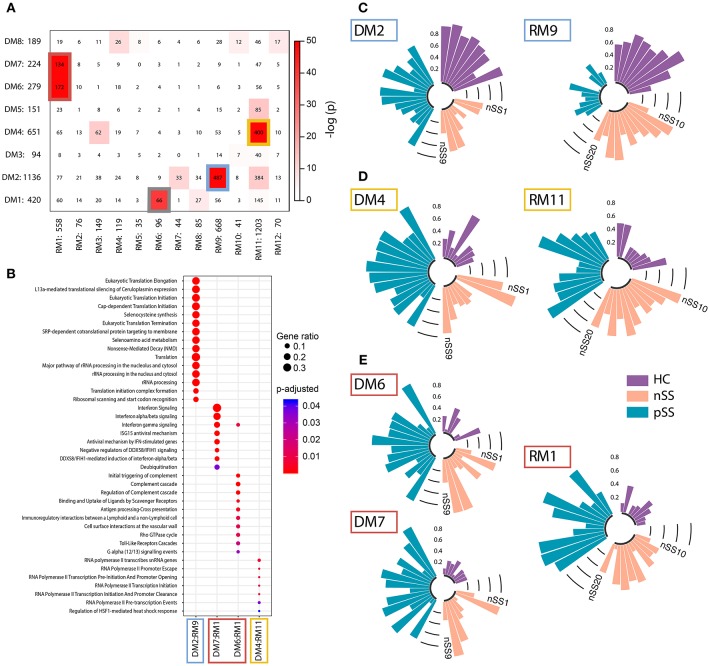
Identification and functional annotation of replicated modules. WGCNA was performed to establish modules of co-expressed genes. To find the most robust and reproducible modules, the extent of overlap between the modules from the discovery and replication analysis was assessed. **(A)** depicts cross-tabulation of discovery modules (DM; rows) and replication modules (RM; columns). Each row and column is labeled by the corresponding module name and the number of replicated genes in the module. Within the table, numbers represent the count of genes present in the intersection of the corresponding row and column module. The table is color-coded by the Fisher exact test *p*-value. Five modules from the discovery cohort exhibited significant overlap with 4 modules from the replication cohort (*p* < 10^−20^). The genes in these replicated modules were designated as gene signatures gray (DM1:RM6), blue (DM2:RM9), yellow (DM4:RM11), and red (DM6+DM7:RM1). Go-term pathway enrichment was used for functional annotation of these signatures, there was no result for the gray signature. Dot-size depicts the fraction of the genes within the pathway that is enriched, color indicates *p*-value of the enrichment **(B)**. Eigengenes (the first principal component) of the blue **(C)**, yellow **(D)**, and red **(E)** signatures are depicted, each bar represents an individual donor and values are in the range [0, 1], where 0 represents the minimum and 1 represents the maximum value.

Genes that share a molecular function have been shown to exhibit correlated changes in their expression profiles (32). To identify the key pathways altered in the patient pDCs, we constructed gene-correlation networks using WGCNA. This method allows the identification of modules of strongly co-expressed genes and their transcriptional hubs. We identified 8 modules from the discovery cohort (DMs) and 12 modules from the replication cohort (RMs) (Supplementary Figure 3). To find the most robust and reproducible modules, we assessed the extent of overlap between the modules from the two cohorts. Five modules from the discovery cohort exhibited significant overlap with four modules from the replication cohort (Figure 3A). The genes in these replicated modules were designated as “gene signatures” gray (DM1:RM6), blue (DM2:RM9), yellow (DM4:RM11), and red (DM6+DM7:RM1). These replicated signatures represent sets of genes that are not only differentially expressed, but also co-expressed in two independent cohorts of patients and controls, emphasizing their consistency and importance.

Functional annotation indicated that the gene signatures were associated with translation and nonsense-mediated decay (blue), transcription initiation and regulation (yellow), and IFN-signaling (red) (Figure 3B). No pathways were enriched for the gray gene signature. Genes from the red and yellow signatures were increased in nSS and pSS patients, the blue signature genes were decreased in patients compared to HC. Again, the nSS patients typically showed gene expression intermediate between the HC and pSS groups (Figures 3C–E, Supplementary Figure 4).

### Identification of Hub-Genes in Identified Signatures

**Figure 4:**
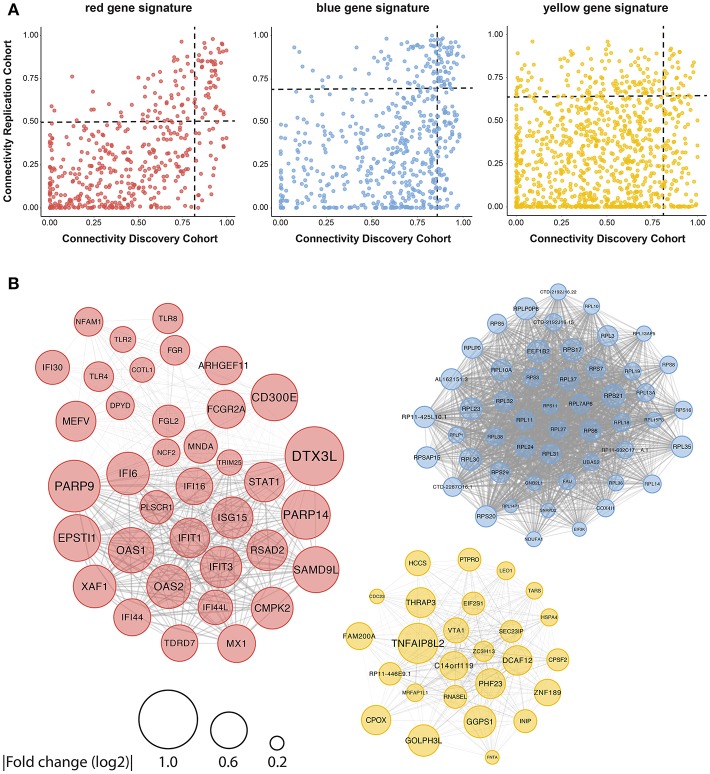
Identification of consistently well-connected hub-genes in identified gene signatures. The connectivity of each gene within the red, blue, and yellow signatures is plotted for both the discovery and replication analysis. For each gene, the connectivity represents the sum of connection strengths with its neighbors in a given module. We normalized the connectivity for each module in the range [0, 1], where 0 represents the minimum and 1 represents the maximum connectivity in the module. Genes with a connectivity above the 75th percentile in both discovery and replication analysis were considered to be hub-genes, the 75th percentile cut-offs for both cohorts are shown with a black dotted line **(A)**. **(B)** The hub genes were plotted based on the data from the replication cohort, the width of the edges between the nodes reflects the strength of their correlation and the size of each node shows the mean fold-change in expression between pSS and HC donors.

To further close in on the most relevant differentially-expressed genes and processes within the signatures, we identified their hub-genes using the normalized connectivity of each gene in both cohorts (Figure 4A). Connectivity indicates the number and strength of correlations each gene has with the other genes in the module. Genes with high connectivity (>75th percentile) in both cohorts were designated as hub and their connections were plotted (Figure 4B, Supplementary Figure 4). Hub-genes in the red signature were involved in signaling pathways downstream of the IFN-α/β receptor and their upregulation was associated with antiviral processes and pDC activation. Genes encoding ribosomal proteins were hub-genes in the blue signature; transcription-regulation genes were hub-genes in the yellow signature. Genes associated with type-I IFN-activity and pDC activation exhibited the largest fold-changes in pSS patients compared to HCs (Figure 4B).

As the red signature hub-genes contained four of the five genes used to quantify the IFN-score (4), we expected this signature to be associated with the IFN-score. To confirm this, we quantified the IFN-score using qPCR on circulating monocytes from 43 of the donors studied (randomly selected). We found a very strong correlation between the monocyte IFN-score and IFIG expression in pDCs, both on the level of individual genes and the composite IFN-score (Supplementary Figure 5A). Thus, the red signature genes indeed strongly correlate with the IFN-score and suggest signaling downstream from the type-I IFN receptor in patient pDCs, associated with pDC activation (9–11). In addition to described IFIGs (19) the red gene signature reveals a large set of novel genes associated with the IFN-signature in pSS pDCs.

### Low Ribosomal Protein Gene Expression Reflects *in vivo* pDC Activation

**Figure 5:**
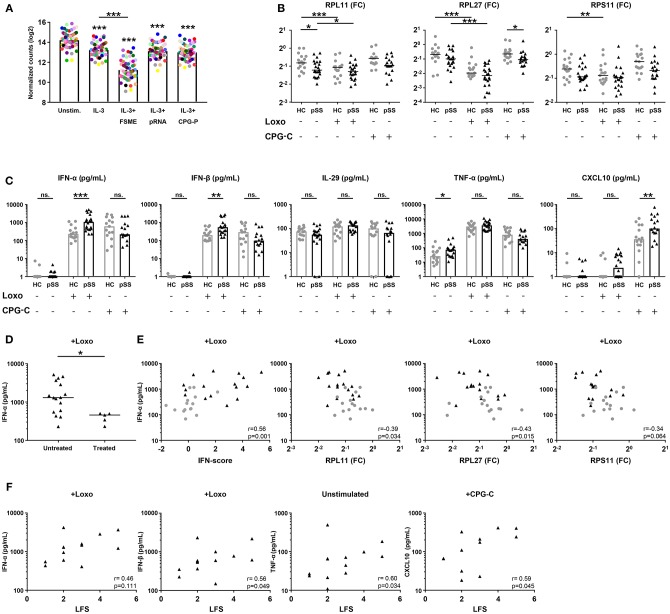
pSS pDCs produce more pro-inflammatory cytokines upon stimulation, associated with expression of hub-genes from the red and blue signatures. Publically available RNA-sequencing data [29] of TLR ligand-stimulated HC pDCs purified from peripheral blood were used to evaluate the effect of pDC activation on ribosomal protein gene-expression. pDCs were stimulated for 6 h in the presence of IL-3 as a survival factor and/or various TLR ligands. Each colored dot represents one specific RP gene, median expression in *n* = 3 donors was plotted for each condition **(A)**. pDCs were purified from peripheral blood of pSS (*n* = 22) and HC (*n* = 17) donors and cultured for 3 h in medium or in the presence of 1 mM loxoribine (Loxo; TLR7L) or 1 μM CPG-C (TLR9L). Supernatants were harvested and cells lysed for mRNA quantification using qPCR. Changes in expression of three major hub-genes from the blue signature were assessed upon stimulation **(B)**. Differences in cytokine secretion between pSS and HC were quantified using luminex **(C)**. Production of IFN-α upon stimulation with loxoribine was compared between patients who were treated with immune-suppressive treatment (treated) and those who were not **(D)**. IFN-α production upon Loxo stimulation in the untreated donors was correlated with the IFN-score, calculated as the mean Z-score of the five quantified IFN-induced genes as compared to HC donors measured in PBMCs, as well as the expression of *RPL11, RPL27*, and *RPS11* measured in the unstimulated condition **(E)**. The association of cytokine production with salivary gland inflammation (measured using the lymphocytic focus score/LFS, which represents the number of lymphocytic aggregates per 4 mm^2^ tissue) in untreated pSS patients was assessed **(F)**. ^*^, ^**^ and ^***^ represent *p* < 0.05, *p* < 0.01, and *p* < 0.001, respectively.

The blue signature represents broadly decreased expression of ribosomal proteins. Classical DCs decrease their transcription and translation of RPs upon stimulation with various activating signals, including TLR ligands (36–38). To investigate the potential link between the decreased expression of RPs in the blue signature and pDC activation, we went back to the RNA sequencing data from the TLR-triggered pDCs (29) and assessed the expression of all expressed RP genes. Stimulation with IL-3 downregulated nearly all RPs, the addition of FSME (primarily a TLR7 ligand) further downregulated the expression of these genes (Figure 5A).

To confirm that activation of pDCs causes broad downregulation of RPs, we cultured pDCs from pSS and HC donors for 3 h in the presence of ligands for TLR7 (loxoribine) or TLR9 (CPG-C) and measured the expression of three hub-genes from the blue gene signature (*RPL11, RPL27, RPS11*). Indeed, all three RPs were downregulated upon stimulation with loxoribine in HC pDCs, while RP expression in pSS patients was typically lower compared to the HC cells consistent with the sequencing data (Figure 5B). Thus, the decreased expression of RPs in the blue signature seems to reflect *in vivo* pDC activation, possibly via TLR7 triggering.

### pSS pDCs Produce Increased Levels of Proinflammatory Cytokines, Correlated With Blue and Red Signature Hub-Genes

Because analysis of their transcriptome indicated that pSS pDCs are activated and/or primed for activation, we set out to confirm this on a functional level. As pro-inflammatory cytokine production is the hallmark of pDC activation, we measured the secretion of cytokines by unstimulated and TLR-triggered pDCs. Upon activation of pSS pDCs with loxoribine, we observed a remarkable mean increase in the production of IFN-α and IFN-β compared to HC. Additionally, pSS pDCs produced more TNF-α in the unstimulated condition and more CXCL10 upon stimulation with CPG-C compared to HC pDCs. No differences were observed for IL-29/IFN-λ1 (Figure 5C) or IL-12 (very low, not shown). Interestingly, we observed significantly lower production of IFN-α and IFN-β upon TLR7 triggering in pSS patients who were on immuno-suppressive treatment at the time of sample collection (Figure 5D, Supplementary Figure 5B).

We next investigated the association between hub-genes from the red and blue gene signatures with cytokine production. As treatment seemed to have an effect on cytokine production, treated patients were excluded from these analyses. Given the fact that the red signature contains the genes typically used to quantify the IFN-signature, we assessed differences in cytokine production between IFN-signature positive and negative patients, but no differences were observed (Supplementary Figure 5C). However, we did observe significant correlations between the continuous IFN-score, reflecting upregulation of the genes in the red signature, and cytokine production for all four conditions where differences between pSS and HC donors were observed (IFN-α and IFN-β upon stimulation with loxoribine; TNF-α without stimulation; CXCL10 with CPG-C). Similarly, the expression of the three blue signature hub-genes (*RPL11, RPL27, RPS11*) correlated with cytokine production (Figure 5E, Supplementary Figure 6). Finally, we observed associations between salivary gland inflammation and cytokine production (Figure 5F), linking circulating pDC dysregulation to local immunopathology.

### Machine Learning Provides Proof of Concept for Harnessing pDC Transcriptional Data as Tool for Patient Stratification

**Figure 6:**
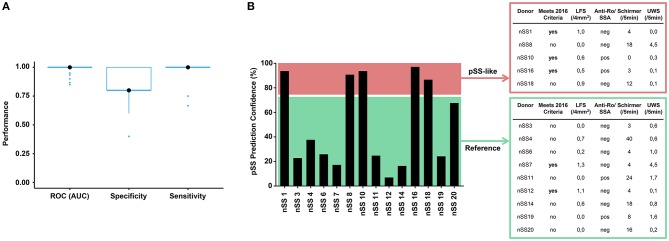
Prediction model discriminates between pSS and HC donors and identifies group of nSS patients more likely to meet the newest classification-criteria. After batch correction of the sequencing data from both cohorts, a selection of 467 genes from the replicated gene signatures was used to build a discriminative classifier that distinguishes between pSS and HC donors. **(A)** shows the performance of the discriminative classifier as validated using 10-fold cross validation. The transcriptional data of the nSS patients from both cohorts were entered into the classifier and a set of “pSS-like” donors (prediction confidence >75%) was identified. nSS patients with lower prediction confidence (<75%) were included as “Reference” group. Fourteen nSS donors from either group were clinically re-evaluated in the context of the new 2016 classification-criteria for pSS at (median [IQR]) 3.5 [2.9–3.9] years after initial sample collection **(B)**.

To assess whether transcriptional data from pDCs could be used for patient stratification, we developed a discriminative classifier using the robust and consistent transcriptional signature of pSS pDCs. Using machine learning, we selected 467 genes from the gene signatures that provided the most discriminative signal between pSS patients and HCs. (Figure 6A, Supplementary Figure 7). This selection allowed us to develop a support-vector machine model with 100% sensitivity and 80% specificity in discriminating between pSS and HCs, underlining their transcriptional dissimilarity. Interestingly, when the nSS data were entered into the model, several donors were designated as “pSS-like” (prediction confidence >75%; *n* = 7).

Patient inclusion was based on the 2002 AECG classification criteria (21) in addition to clinical evaluation by an experienced rheumatologist. After sample collection for this study had finished, new Sjögren's syndrome classification-criteria were established with improved sensitivity and suitability for detection of patients at early stages of disease (22, 39). In this context, we wondered whether part of the nSS patients that our prediction model designated as pSS-like would meet these new classification-criteria at follow-up. For this, we invited all twenty included nSS donors for clinical re-evaluation, to which fourteen patients agreed. Five pSS-like donors and nine of the other nSS patients (“Reference” group; <75% prediction confidence) were clinically re-evaluated at (median [IQR]) 3.5 [2.9–3.9] years after initial sample collection. At follow-up, three of the pSS-like patients (60%) met the new pSS classification-criteria; this was true for only two of the donors from the reference group (22%) (Figure 6B), suggesting that pDC transcriptional data may hold promise for patient stratification.

## Discussion

We here exploited RNA-sequencing to provide in-depth transcriptional analysis of pDCs from patients with pSS and nSS. The use of two independent cohorts of patients and controls allowed us to define robustly reproducible signatures of consistently altered and co-expressed genes. This included the differentially expressed genes directly related to the IFN-signature and those independent of it. Using this approach, we corroborate the majority of previously reported transcriptional alterations in pDCs from a smaller cohort of IFN-signature positive pSS patients (19). Relevant exceptions include *MYD88, TLR7*, and *TLR9*; we did observe clear correlations of *TLR7* and *MYD88* with the IFN-score in both cohorts, supporting the published data (19), but we did not observe evidence for *TLR9* downregulation in pSS patients or IFN-signature positive patients. Importantly, our data greatly expand upon the existing literature and identify a large set of novel genes associated with the IFN-signature in pSS pDCs. Some of these genes (e.g., *DTX3L, PLSCR1, FCGR2A*) are top hits in published studies in pSS (40–42). *FCGR2A* is of particular interest, as it is associated with internalization of immune complexes by pDCs (43, 44), which is a likely pathway by which pDCs are activated in pSS. Others are known IFN-induced genes that were not yet described in pSS (e.g., *PARP14, TDRD7*) with functions in anti-viral immunity and regulation of type-I IFN production (45, 46). In addition, the red gene signature includes *TLR2, TLR4*, and *TLR8*, which are not prominent pDC receptors (8) but may play a role in pDC activation under specific conditions, such as during viral infection or in inflamed tissues (47–49).

The yellow gene signature importantly contains *CCR5*, which is a chemokine receptor that is important for effective migration of pDCs to inflamed tissues and lymphnodes (35, 50, 51). We confirmed the upregulation of CCR5 in pSS pDCs on the protein level and in view of the increased levels of its ligands CCL3 and CCL4 in saliva of pSS patients (52), this receptor may mediate the migration of pDCs toward the salivary glands in pSS (15, 17, 18). Furthermore, the yellow signature contains several hub genes involved in the regulation of apoptosis (e.g., *DCAF12, TNFAIP8L2*) as well as the transcription factors *SP1* and *TP53*, which are central regulators of this process (53–55). The aberrant regulation of apoptosis may contribute to the decreased numbers of pDCs in the blood of patients with pSS (15). For the gray signature, functional annotation using pathway enrichment did not indicate any specific function. As pathway enrichment only considers known processes, novel data on these genes may shed more light on the function of the gray signature in the future.

The blue gene signature contains the majority of ribosomal protein genes as well as a range of ribosomal co-factors and is primarily involved in translation. To our knowledge, this is the first study that describes a broadly decreased expression of ribosomal proteins in pDCs in any setting. Using a published dataset (29) and *in vitro* pDC cultures, we show that this broad RP downregulation is associated with pDC activation via TLR7. In addition to translation, RPs have a broad range of cellular functions, including regulation of cytokine signaling and the preferential translation of specific (viral) transcripts (56–60). Furthermore, this signature is associated with nonsense-mediated decay, which is an important quality-control mechanism that prevents disease by blocking translation of faulty transcripts and regulates important cellular processes by targeting intact mRNA (61). As such, this gene signature potentially has far-reaching consequences on pDC function. Interestingly, downregulation of RPs can be used by antigen-presenting cells to increase their relative abundance of exogenous antigen, and thus promote cross-presentation of viral peptides rather than endogenous peptides to CD8 T cells (38), which may be an ongoing process in pSS pDCs. The fact that TLR7L stimulation enables pDCs to effectively (cross-) present antigen (62, 63) corroborates the link between broad downregulation of RPs observed upon TLR ligation and cross-presentation of antigen. Future studies that combine transcriptomic data with proteomic data should shed light on the full extent of RP dysregulation in pSS pDCs.

On the basis of [1] gene-set enrichment data indicating that patient pDCs are similar to TLR-triggered pDCs; [2] the downregulation of RP genes upon pDC activation, mimicking the blue signature; [3] evidence of signaling downstream of the type-I IFN receptor in the red signature, we conclude that pDCs from patients with pSS, and to a lesser extent nSS, have an activated phenotype at the gene expression level. As stimulation with even very low levels of type-I IFN results in enhanced cytokine production by pDCs upon TLR triggering and is required for optimal pDC activation (9–11), we hypothesized that patient pDCs are primed for enhanced cytokine production. To functionally validate this, we activated pDCs via TLR7 and TLR9 in a short culture of 3 h to minimize any effects of secreted type-I IFNs [produced after ~2 h of culture (10)] on the transcriptome of the cultured cells. Indeed, pSS pDCs produced markedly higher type-I IFN levels, which correlated with the IFN-score and the expression of hub-genes from the blue signature, indicating that their transcriptome underpins the enhanced cytokine production. Interestingly, treatment was associated with reduced type-I IFN production by pDCs and seemed to affect the pDCs at the transcriptional level as well, as three of the four pSS patients misclassified in the prediction model were treated at time of sampling. Hydroxychloroquine was the most often used drug in these cohorts, and these findings are in-line with its effects on circulating immune cells as well as cultured cells described in literature (19, 64, 65).

The activated phenotype and enhanced production of pro-inflammatory cytokines by pSS pDCs can substantially affect salivary gland inflammation. Type-I IFNs promote B cell-hyperactivity by driving B cell expansion, migration, and differentiation (4, 18, 66, 67). In addition, type-I IFNs stimulate lymphocyte attraction and expansion via activation of classical dendritic cells and other immune cells (68–70). The correlation between pDC cytokine production and the lymphocytic focus score corroborates the relevance of the altered pDC function for local inflammation. Considering the increased expression of CCR5 on pSS pDCs and the presence of its ligands CCL3 and CCL4 in saliva of pSS patients (52), this receptor may act as the link between circulating pDCs and the salivary glands in pSS (15, 17, 18).

nSS patients form a poorly studied patient group with similar levels of dryness compared to pSS patients, but limited signs of (local) autoimmunity. To examine whether pDC dysregulation may play a role in these patients we included them in this study. Our data indicate that pDCs from nSS patients are similarly but less strongly activated compared to those from pSS patients on a group level. A set of nSS patients, who all did not meet the 2002 classification-criteria (21) at initial sample collection, was defined as pSS-like by our discriminative model and a relatively large fraction of them met the newest classification-criteria (22) at follow-up. A limitation of this part of the study is that we were unable to include ocular staining in our clinical assessment. In addition, changes may have occurred in these patients in the time-period between initial sampling and follow-up. For instance, an increased focus score was observed in several donors compared to baseline. However, the relative effects of the enhanced sensitivity of the new criteria and any evolution of clinical manifestations are difficult to estimate. Nevertheless, our model discriminates between pSS and HC donors with high accuracy and additionally identifies a group of nSS patients of whom a relatively large fraction meets the more sensitive 2016 criteria at follow-up. As such, we provide proof of concept for further exploration of pDC transcriptional data as potential tool for patient stratification in sicca patients in future studies.

Concluding, we show that pSS pDCs are robustly distinct from HC pDCs, have an activated transcriptional phenotype, and are primed for increased pro-inflammatory cytokine production. nSS patients form an intermediate group in which some patients are molecularly similar to pSS patients in concordance with the most recent classification-criteria. In addition, the expression of hub genes from the two major identified gene signatures correlates with cytokine production, indicating that the transcriptional dysregulation of pSS pDCs underpins their enhanced cytokine production. These data substantiate the notion that pDCs play a role in pSS immunopathology and support studies that target pDCs, type-I IFNs, or IFN-signaling in pSS.

## Data Availability

The RNA sequencing datasets generated for this study can be found in the GEO database at https://www.ncbi.nlm.nih.gov/geo/ - GSE135635. The raw data supporting the conclusions of this manuscript will be made available by the authors, without undue reservation, to any qualified researcher.

## Ethics Statement

The medical ethics committee of the UMC Utrecht approved the study (METC; nr 13-697), all patients gave their written informed consent.

## Author Contributions

MH, AP, SB, MR, AK, JR, and TR were involved in conception and design of the study. MH, SB, SH, CB, EH, and AK were involved in data acquisition. MH, AP, SB, NS, AK, JR, and TR were involved in data analysis and interpretation. MH and AP drafted the manuscript. All authors revised the manuscript critically for important intellectual content and approved the submitted version.

### Conflict of Interest Statement

TR is a principal investigator in the immune catalyst program of GlaxoSmithKline, which is an independent research program. He did not receive any financial support other than the research funding for the current project. The remaining authors declare that the research was conducted in the absence of any commercial or financial relationships that could be construed as a potential conflict of interest.
