# Supplementary material for: Plasmacytoid DCs From Patients With Sjögren's Syndrome Are Transcriptionally Primed for Enhanced Pro-inflammatory Cytokine Production
Source: Front Immunol. 2019 Sep 4;10:2096. doi: 10.3389/fimmu.2019.02096 (PMC6736989; doi:10.3389/fimmu.2019.02096)
Supplement: Supplementary file 1 [file Data_Sheet_1.PDF]

1 Supplementary Figures and Tables

Supplementary Table 1. List of primers used

Gene	Primer	Sequence
RPL11	Fw	AAGGGTCTAAAGGTGCGGGA
	Rv	CACATAGAAGTCCAGGCCGT
RPL27	Fw	GGGGTGAAAGGTTAGCGGAAG
	Rv	CCCATTTCGGCAGCAACCAC
RPS11	Fw	CTGAGCGTGCCTACCAAAAG
	Rv	CTCAATAGCCTCCTTGGGTGT
Ly6E	Fw	ATCTGTACTGCCTGAAGCCG
	Rv	GTCACGAGATTCCCAATGCC
IFIT3	Fw	ACTGTTTCAACGGGTGTTGG
	Rv	CCTTGTAGCAGCACCCAATC
IFI44L	Fw	CCACCGTCAGTATTTGGAATGT
	Rv	ATTTCTGTGCTCTCTGGCTT
IFI44	Fw	TTTGCTCTTTCTGACATCTCGGT
	Rv	TCCTCCCTTAGATTCCCTATTTGC
MX-1	Fw	GCATCCCACCCTCTATTACTG
	Rv	CGCACCTTCTCCTCATACTG
GUSB	Fw	CACCAGGGACCATCCAATACC
	Rv	GCAGTCCAGCGTAGTTGAAAAA
GAPDH	Fw	ATGGGGAAGGTGAAGGTCG
	Rv	GGGGTCATTGATGGCAACAATA

Supplementary Table 2. List of FACS antibodies used.

Experiment	Antibody	Fluorochrome	Company
Monocyte purity	anti-CD14	FITC	Miltenyi
	anti-CD16	PE	Agilent
	anti-CD45	PerCP	Sony
pDC purity	anti-CD3	APC	BD
	anti-CD14	APC-eF780	eBioscience
	anti-CD19	BV421	Sony
	anti-CD45	PerCP	Biolegend
	anti-CD123	PE	Miltenyi
	anti-BDCA-2	FITC	Miltenyi
CCR5 Expression	anti-CD3	AF700	Sony
	anti-CD4	APC-eF780	eBioscience
	anti-CD8	V500	BD
	anti-CD14	BV785	Biolegend
	anti-CD16	APC	eBioscience
	anti-CD19	PE-Cy7	BD
	anti-CD56	PE-CF594	BD
	anti-CD123	FITC	Biolegend
	anti-HLA-DR	BV605	BD
	anti-BDCA-2	PerCP-Cy5.5	Sony
	anti-CCR5	PE	BD

1.1 Supplementary Figures

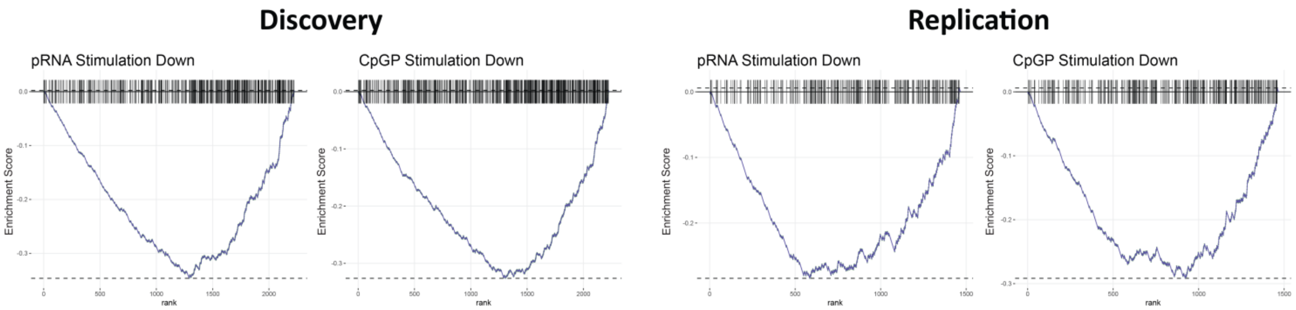

Supplementary Figure 1. Comparison of the downregulated genes in pSS pDCs and TLR-triggered pDCs. Using gene-set enrichment analysis, we compared the transcriptional profile of pSS pDCs with that of *in vitro* stimulated pDCs. There was a large overlap in downregulated genes between pDCs stimulated with either pRNA (TLR7 ligand) or CPG-P (TLR9 ligand) and those from pSS pDCs (all FDR-corrected $p<0.05$).

Supplementary Figure 2 (next page). Most genes previously shown to be increased in pDCs from IFN-positive pSS patients are also expressed at a higher level at group level in pSS. Expression of Toll-like receptors and genes increased in IFN-signature positive patients from both analyzed cohorts. HC are depicted in purple, nSS patients in pink, and pSS patients in green. *, **, and *** depicted p-values of <0.05, <0.01, and <0.001, respectively.

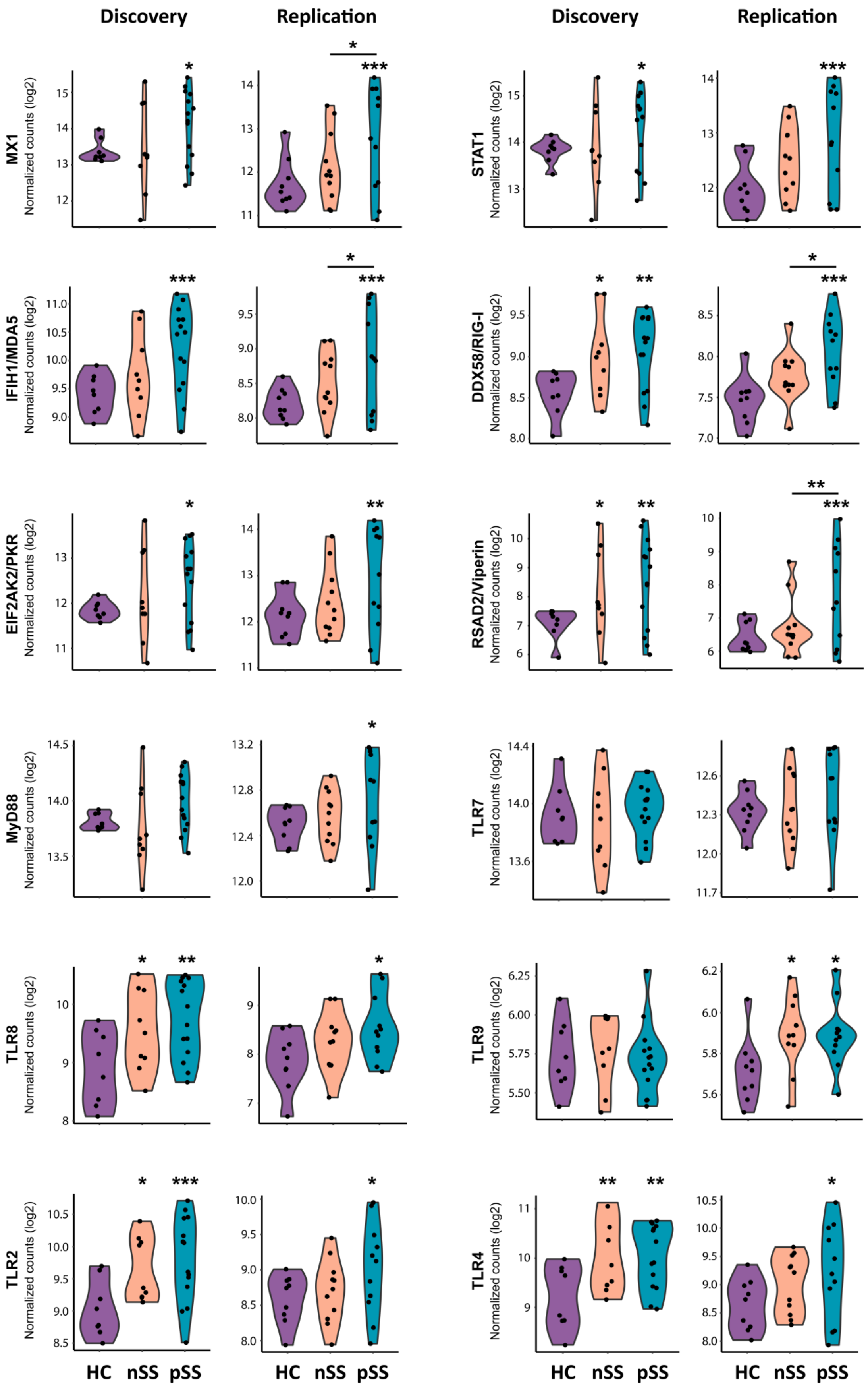
Discovery
Replication
Discovery
Replication
MX1
STAT1
IFIH1/MDA5
DDX58/RIG-I
EIF2AK2/PKR
RSAD2/Viperin
MyD88
TLR7
TLR8
TLR9
TLR2
TLR4
Normalized counts (log2)
HC
nSS
pSS

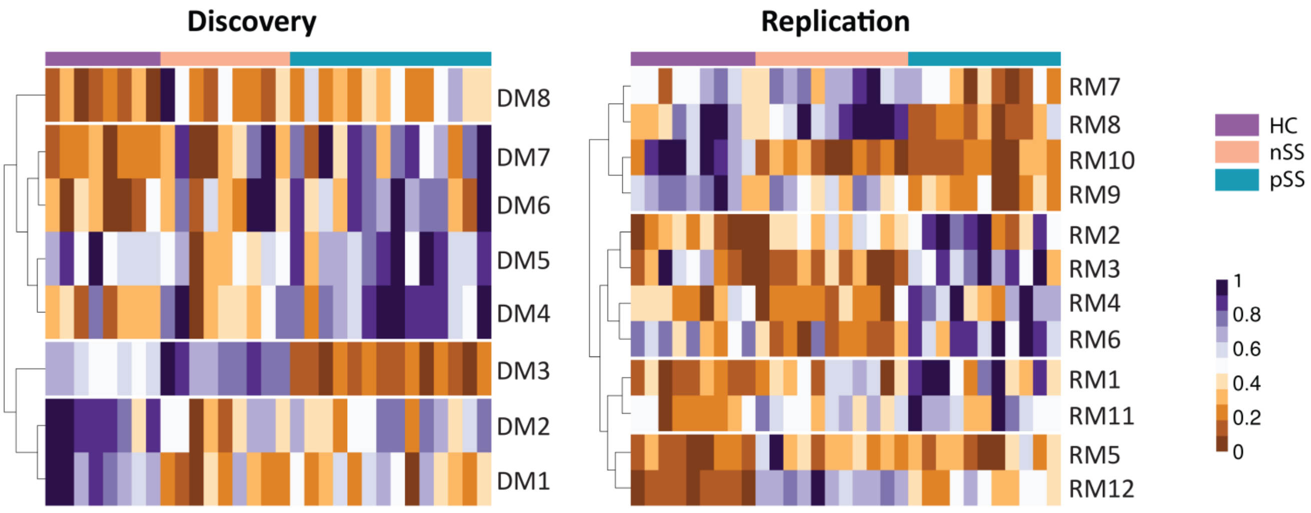

Supplementary Figure 3. Unsupervised clustering of modules from discovery and replication cohorts. Modules from both cohorts were clustered based on the eigengene value in each donor. Each column represents an individual donor and values are in the range [0, 1], where 1 represents the maximum and 0 represents the minimum eigengene value.

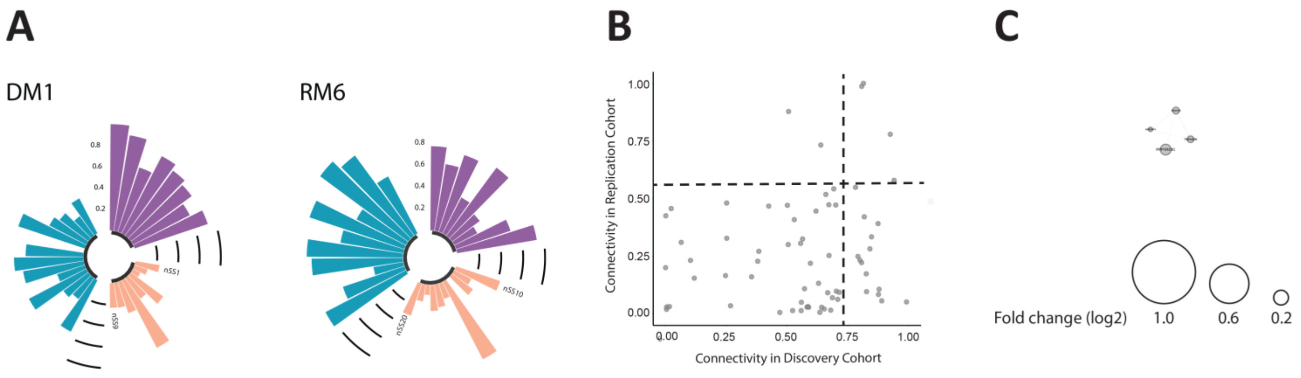

Supplementary Figure 4. Gray signature characteristics. Eigengene values for the modules within the gray gene signature in each cohort (A). Each bar represents an individual donor and values are in the range [0, 1], where 1 represents the maximum and 0 represents the minimum eigengene value. The connectivity of each gene within the gray signature is plotted for both the discovery and replication cohorts. We normalized the connectivities for each module in the range [0, 1] (B). Genes with a connectivity within the 75th percentile in both discovery and replication analysis were considered to be hub-genes and were plotted (C). Based on the data from the replication cohort, the width of the edges between the nodes reflects the strength of their correlation and the size of each node is a reflection of the mean fold-change in expression between pSS and HC donors.

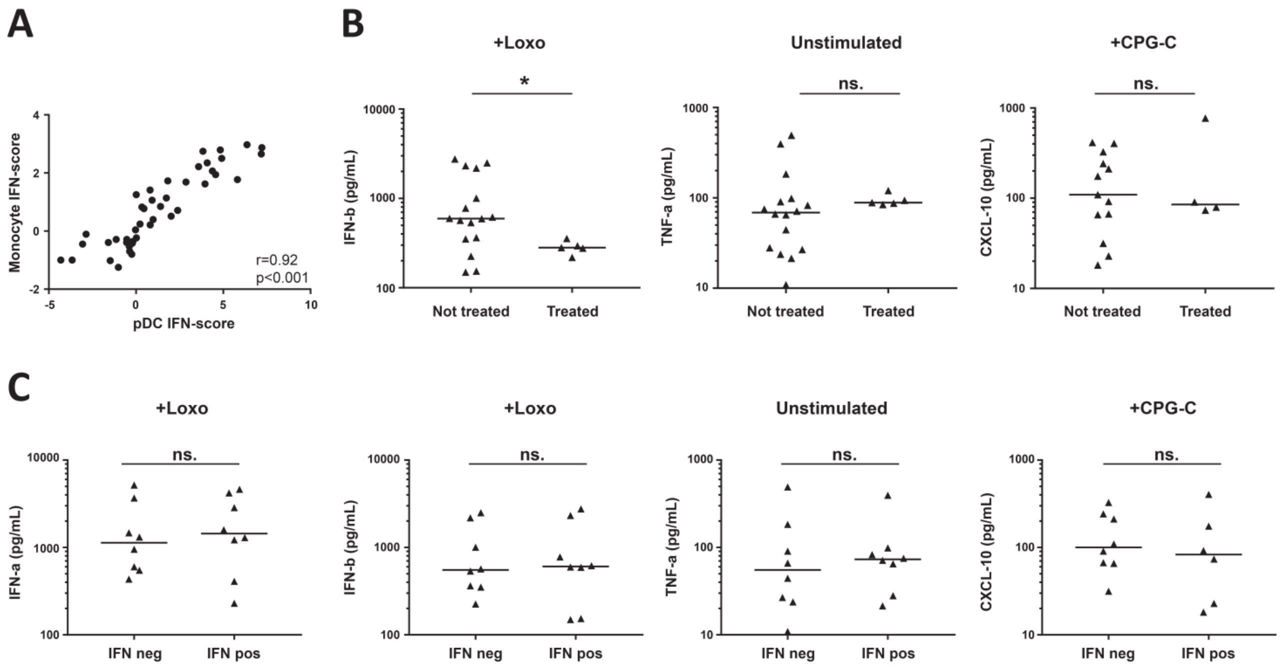

Supplementary Figure 5. Treatment is associated with decreased type-I IFN production by cultured pDCs. Correlation of the IFN-score quantified in purified monocytes (by qPCR) and pDCs (by RNAseq) from the same donor. Score was calculated as the mean Z-score of the five quantified IFN-induced genes as compared to the respective HC donors (A). Purified pDCs were isolated from the circulation of pSS and HC donors and cultured for 3 hours in the presence or absence of ligands for TLR7 (Loxoribine) or TLR9 (CPG-C). Supernatants were harvested and cytokines were measured using Luminex. For selected conditions, production of cytokines was compared between pSS patients that were being treated with immunosuppressive drugs at time of sampling (treated) or not (B). The IFN-score was quantified in PBMCs using qPCR and patients with an IFN-score≥2.0 were defined as being IFN-signature positive (IFN pos). Cytokine production by cultured pDCs was compared between IFN-neg and IFN-pos patients for all conditions for which significant differences were observed between pSS and HC (C). Spearman's rank coefficient and corresponding p-values are shown for correlations, differences between groups were analyzed using Mann-Whitney U-test. * represents $p<0.05$.

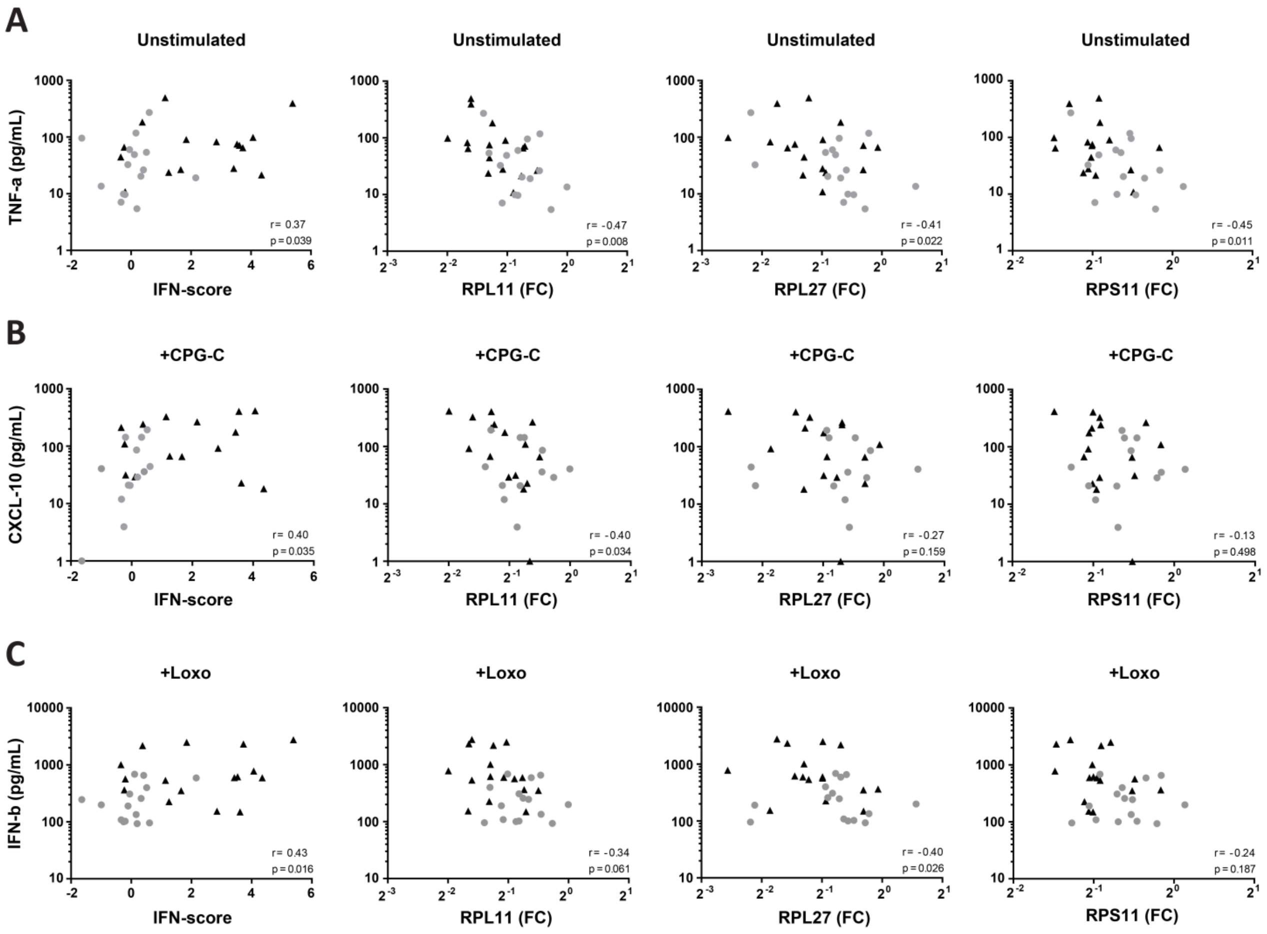

Supplementary Figure 6. Genes from blue and red signatures correlate with cytokine production by cultured pDCs. Purified pDCs were isolated from the circulation of pSS and HC donors and cultured for 3 hours in the presence or absence of ligands for TLR7 (Loxoribine) or TLR9 (CPG-C). Supernatants were harvested and cytokines were measured using Luminex, cells were lysed and gene expression was quantified using qPCR. IFN-score was measured in paired PBMCs. For all conditions for which a significant difference between pSS and HC was observed, cytokine production was correlated with IFN-score and expression of ribosomal protein genes from blue signature (A-C). Spearman's rank coefficient and corresponding p-values are shown.

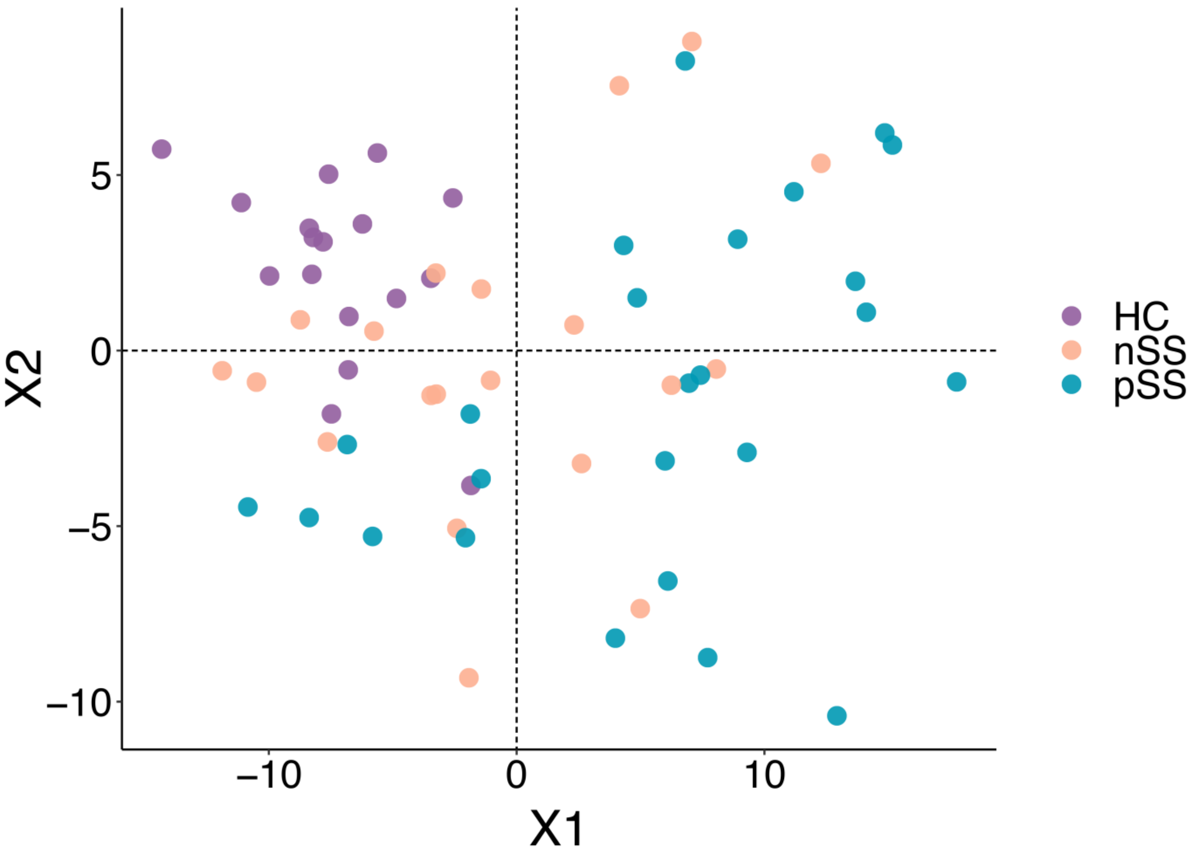

Supplementary Figure 7. Multi-dimensional scaling plot of donors based on genes included in discriminative classifier. A selection of 467 genes from the replicated gene signatures was used to build a discriminative classifier (Figure 6) that distinguishes between pSS and HC donors. Multidimensional scaling shows that the selected replicated genes can distinguish between pSS and HC.
